# Protective Effect of Glutathione against Oxidative Stress-induced Cytotoxicity in RAW 264.7 Macrophages through Activating the Nuclear Factor Erythroid 2-Related Factor-2/Heme Oxygenase-1 Pathway

**DOI:** 10.3390/antiox8040082

**Published:** 2019-04-01

**Authors:** Da Hye Kwon, Hee-Jae Cha, Hyesook Lee, Su-Hyun Hong, Cheol Park, Shin-Hyung Park, Gi-Young Kim, Suhkmann Kim, Heui-Soo Kim, Hye-Jin Hwang, Yung Hyun Choi

**Affiliations:** 1 Anti-Aging Research Center, Dong-eui University, Busan 47340, Korea; 13956@deu.ac.kr (D.H.K.); 14769@deu.ac.kr (H.L.); hongsh@deu.ac.kr (S.-H.H.); 2 Department of Parasitology and Genetics, Kosin University College of Medicine, Busan 49267, Korea; hcha@kosin.ac.kr; 3 Department of Biochemistry, Dong-eui University College of Korean Medicine, Busan 47227, Korea; 4 Department of Molecular Biology, College of Natural Sciences, Dong-eui University, Busan 47340, Korea; parkch@deu.ac.kr; 5 Department of Pathology, Dong-eui University College of Korean Medicine, Busan 47227, Korea; omdpark@deu.ac.kr; 6 Department of Marine Life Sciences, Jeju National University, Jeju 63243, Korea; immunkim@jejunu.ac.kr; 7 Department of Chemistry, College of Natural Sciences, Center for Proteome Biophysics and Chemistry Institute for Functional Materials, Pusan National University, Busan 46241, Korea; suhkmann@gmail.com; 8 Department of Biological Sciences, College of Natural Sciences, Pusan National University, Busan 46241, Korea; khs307@pusan.ac.kr; 9 Department of Food and Nutrition, College of Nursing, Healthcare Sciences & Human Ecology, Dong-eui University, Busan 47340, Korea; hhj2001@deu.ac.kr

**Keywords:** glutathione, oxidative stress, DNA damage, apoptosis, ROS, Nrf2/HO-1

## Abstract

Reactive oxygen species (ROS), products of oxidative stress, contribute to the initiation and progression of the pathogenesis of various diseases. Glutathione is a major antioxidant that can help prevent the process through the removal of ROS. The aim of this study was to evaluate the protective effect of glutathione on ROS-mediated DNA damage and apoptosis caused by hydrogen peroxide, H_2_O_2_, in RAW 264.7 macrophages and to investigate the role of the nuclear factor erythroid 2-related factor-2 (Nrf2)/heme oxygenase-1 (HO-1) signaling pathway. The results showed that the decrease in the survival rate of RAW 264.7 cells treated with H_2_O_2_ was due to the induction of DNA damage and apoptosis accompanied by the increased production of ROS. However, H_2_O_2_-induced cytotoxicity and ROS generation were significantly reversed by glutathione. In addition, the H_2_O_2_-induced loss of mitochondrial membrane potential was related to a decrease in adenosine triphosphate (ATP) levels, and these changes were also significantly attenuated in the presence of glutathione. These protective actions were accompanied by a increase in the expression rate of B-cell lymphoma-2 (Bcl-2)/Bcl-2-associated X protein (Bax) and poly(ADP-ribose) polymerase cleavage by the inactivation of caspase-3. Moreover, glutathione-mediated cytoprotective properties were associated with an increased activation of Nrf2 and expression of HO-1; however, the inhibition of the HO-1 function using an HO-1 specific inhibitor, zinc protoporphyrin IX, significantly weakened the cytoprotective effects of glutathione. Collectively, the results demonstrate that the exogenous administration of glutathione is able to protect RAW 264.7 cells against oxidative stress-induced mitochondria-mediated apoptosis along with the activity of the Nrf2/HO-1 signaling pathway.

## 1. Introduction

Although adequate levels of reactive oxygen species (ROS) act as signaling molecules that are essential for cell growth and proliferation, the increased production of ROS can cause oxidative damage to cells [1,2]. Because mitochondria are one of the major intracellular organelles of ROS production and are the most vulnerable targets of ROS, an inadequate accumulation of ROS due to oxidative stress has been recognized as one of the mechanisms leading to apoptosis following DNA damage associated with mitochondrial dysfunction [3,4,5]. The increase in ROS levels is due to changes in the intracellular redox balance of cells, and the failure of antioxidant mechanisms to eliminate ROS production can promote this [6]. Moreover, the accumulation of ROS beyond the antioxidant function of cells could reduce the mitochondrial membrane potential (MMP), an index of the electron transport chain performance, resulting in a compromised adenosine triphosphate (ATP) production [4,7]. Subsequently, apoptogenic factors such as cytochrome *c* are released into the cytoplasm from the mitochondrial intermembrane space due to the loss of MMP, and the caspase cascade is activated, which could eventually trigger apoptosis. Ultimately, intracellular ATP levels can also be used as an important index for assessing the homeostasis of the mitochondrial energy metabolism associated with oxidative stress [8,9]. Although cells have various endogenous antioxidant protection systems to protect against the harmful effects of ROS, these enzymes cannot effectively eliminate excessive ROS generation. Therefore, a supplementation of antioxidants has been proposed as a strategy to prevent free radical accumulation through the activity of the signal pathway corresponding to the ROS production.

Glutathione (GSH) is a ubiquitous thiol-containing tripeptide that consists of L-cysteine, L-glutamic acid, and glycine, and it is one of the most studied cell antioxidants currently being researched [10,11,12,13]. Many antioxidants used to block oxidative stress are chemically converted into oxidation products that react with glutathione to form glutathione adducts during the protection against free radicals [14,15]. These antioxidant defense effects of glutathione seem to play an important role in regulating cell proliferation and cell death through the mediation of the main redox regulatory signaling pathway in the cell [10,16]. Previous studies have also shown that the supply of glutathione prevents cell damage due to oxidative stress, while the loss of the glutathione-dependent enzyme pathway due to reduced glutathione levels contributes to the onset and progression of many diseases [15,17]. In humans, GSH is the most abundant non-protein intracellular thiol present in 1-10 mM concentrations. However, a number of different pathological conditions are also associated with dysregulated GSH synthesis or alterations in its concentration, which can have potential applications as a marker for human disease [18].

Recently, the activation of nuclear factor erythroid 2-related factor-2 (Nrf2), a leucine zipper redox-sensitive transcriptional regulator, has been reported to be involved in mitigating cellular damage induced by oxidative stress [19,20]. Upon exposure to oxidative stress, Nrf2 is translocated from the cytoplasm into the nucleus, where it results in the transcriptional activation of antioxidant and detoxification genes. Since Nrf2 must be dissociated from kelch-like ECH-associated protein-1 (Keap1) in order to migrate to the nucleus, Keap1 acts as an inhibitor of Nrf2, which retains the Nrf2 protein in the cytoplasm and prevents the transcription of downstream target genes [20,21]. As one of the Nrf2-dependent cytoprotective enzymes, heme oxygenase-1 (HO-1) acts as an important rate-limiting enzyme in the metabolic conversion of heme to the bile pigments, such as biliverdin and bilirubin, and thus constitutes a major intracellular source of iron and carbon monoxide. This enzyme is also activated in response to various oxidative signals and provides adaptive and beneficial cellular responses to oxidative damage [21,22]. The fact that HO-1 acts as a cytoprotective enzyme has been suggested by observing that the increased expression of HO-1 can inhibit the formation of ROS due to oxidative stress [20,21,22]. Therefore, HO-1 provides an adaptive cellular response to oxidative damage and is not limited to the degradation of toxic heme released by hemoproteins. In addition, HO-1 has been shown to further support the cellular protective role of this gene by inhibiting a variety of inflammatory responses including endotoxin shock [21,22,23]. Although there is diverse evidence that Nrf2/HO-1 signaling protects against cell death by preventing excessive ROS generation under various oxidative stress conditions, evidence that its signaling is involved in overcoming oxidative stress by glutathione has not been well researched to date. Therefore, in the present study, we investigated whether glutathione ameliorates oxidative stress (hydrogen peroxide, H_2_O_2_)-induced mitochondria-mediated apoptosis and whether the Nrf2/HO-1 signaling pathway is involved in this process in RAW 264.7 macrophages.

## 2. Materials and Methods

### 2.1. Cell Culture and Glutathione Treatment

RAW 264.7 cells were purchased from the Korea Cell Line Bank (Seoul, Republic of Korea). Cells were cultured in Dulbecco’s Modified Eagle’s Medium (DMEM) supplemented with 10% fetal bovine serum, 2 mM L-glutamine and 100 U/mL penicillin/streptomycin (all from WelGENE Inc., Daegu, Republic of Korea) at 37 °C in a humidified 5% CO_2_ atmosphere. Glutathione (reduced) was purchased from Daehan New Pharm Co. (Hwasung, Republic of Korea), dissolved in dimethyl sulfoxide (pH 7.4, DMSO; Sigma-Aldrich Chemical Co., St. Louis, MO, USA) and, prior to use in the experiments, diluted with a cell culture medium to adjust the final treatment concentrations (final concentration of DMSO, <0.05%). DMSO (<0.05%) alone did not have any effect on the parameters measured.

### 2.2. Cell Viability Assay

For the cell viability study, RAW 264.7 cells were seeded at 200 μL in a 96-well plate at a density of 5 × 10^3^ cells per well. After a 24 h incubation, the cells were incubated with the control medium (0.05% DMSO) or different concentrations of H_2_O_2_ for another 24 h or pre-incubated with various concentrations of glutathione for 1 h before a 500 μM H_2_O_2_ treatment for 24 h. The cells were also treated with 10 μM zinc protoporphyrin IX (ZnPP; Sigma-Aldrich Chemical Co.), a well-established HO-1 inhibitor, for 1 h in the presence or absence of H_2_O_2_. Subsequently, 3-(4,5-dimethylthiazol-2-yl)-2,5-diphenyltetrazolium bromide (MTT; Sigma-Aldrich Chemical Co.) solution (0.5 mg/mL) was added to each well incubated at 37°C for 3 h. After removing the medium, 200 µL of DMSO were added to each well to dissolve the formazan for 10 min. The effect for cell viability was assessed by measuring the optical density at a wavelength of 560 nm with a microplate reader (Dynex Technologies, Chantilly, VA, USA). The experiment was repeated in triplicate and the cell viability was determined by dividing the absorbance values of the treated cells to that of untreated (control) cells.

### 2.3. Apoptosis Assay Using a Fluorescence Microscope

In order to evaluate the induction of apoptosis by observing the morphological changes of nuclei, the cells were harvested and washed with phosphate buffered saline (PBS) and then fixed with 4% paraformaldehyde (Sigma-Aldrich Chemical Co.) in PBS for 30 min at room temperature. Next, the cells were permeabilized with 0.1% (*w/v*) Triton X-100 for 5 min and stained with a solution of 1.0 mg/mL 4,6-diamidino-2-phenylindole (DAPI; Sigma-Aldrich Chemical Co.) for 10 min at room temperature in the dark, before being washed twice with PBS. The morphology changes in the nucleus were examined using a fluorescence microscope (Carl Zeiss, Oberkochen, Germany), at an excitation wavelength of 340 nm and an emission wavelength of 488 nm.

### 2.4. Apoptosis Analysis using a Flow Cytometer

The extent of apoptosis was determined with a flow cytometer using annexin V/propidium iodide (PI) double staining. In brief, the cells were resuspended in the supplied binding buffer and then stained with fluorescein isothiocyanate (FITC)-conjugated annexin V and PI (BD Pharmingen, San Diego, CA, USA) at room temperature for 20 min without light, according to the manufacturer’s protocol. The fluorescent intensities of the cells were detected with a flow cytometer (Becton Dickinson, San Jose, CA, USA), and the acquisition was performed using Cell Quest Pro software (Becton Dickinson). A schematic plot was used to display the results: the lower left quadrant represents live cells; the lower right and upper right quadrants represent early and late apoptotic cells, respectively; and the upper left quadrant represents necrotic cells. Apoptosis refers to the sum of early and late apoptotic cells.

### 2.5. Colony Formation Assay

After the treatment with H_2_O_2_ for 24 h in the presence or absence of glutathione, a total of 500 cells per well were seeded in a 6-well plate in triplicate and maintained in a humidified atmosphere containing 5% CO_2_ at 37 °C. After being cultured for two weeks, the colonies were washed with PBS, fixed with 3.7% paraformaldehyde for 30 min and stained with 0.1% purple-violet solution (Sigma-Aldrich Chemical Co.) for 10 min. After washing by PBS, the colonies containing more than 50 cells were counted and photographed under an inverted microscope (Carl Zeiss).

### 2.6. HO-1 Activity Assay

In order to measure the HO-1 enzyme activity, lysates of cells were prepared according to a previous study [24], and homogenates containing biliverdin reductase were obtained from rat liver. The lysates and the homogenates were treated with nicotinamide adenine dinucleotide phosphate and hemin for 1 h, while blank samples contained only hemin. The concentration of bilirubin degraded by HO-1 from hemin was determined as the difference in absorbance at 464 and 530 nm using a microplate reader. The HO-1 activity was expressed as picomoles of bilirubin per milligram of protein.

### 2.7. Measurement of ROS Level

To measure the formation of intracellular ROS, RAW 264.7 cells were seeded onto 6-well plates with a density of 3 × 10^5^ cells per well for 24 h and treated with or without glutathione for 1 h before adding H_2_O_2_ for a further 1 h. The cells were washed twice with PBS, suspended in PBS, and stained with 10 μM of 2′,7′-dichlorofluorescein diacetate (DCF-DA; Sigma-Aldrich Chemical Co.) for 20 min at 37 °C, away from light. The relative fluorescent intensity of the cell suspensions was measured with a flow cytometer (Becton Dickinson). For the image analysis of the intracellular ROS production, the cells were seeded on coverslip-loaded 6-well plates. After 24 h of plating, the cells were treated with glutathione, and 1 h later, H_2_O_2_ was added to the plate for 1 h. After washing with PBS, 10 μM DCF-DA was loaded into the wells and incubated at 37 °C for an additional 20 min. The stained cells were washed and mounted on a microscope slide using a mounting medium (Sigma-Aldrich Chemical Co.), and the images were visualized using a fluorescence microscope at an excitation wavelength of 485 nm and an emission wavelength of 530 nm.

### 2.8. Western Blot Analysis

After being subjected to the necessary experimental treatments, the cells were harvested, washed with PBS, and lysed with a lysis buffer for 30 min to extract whole-cell proteins, as described in the previous study [25]. The cytosolic and nuclear proteins were extracted using an NE-PER™ Nuclear and Cytoplasmic Extraction Reagents (Thermo Fisher Scientific, Waltham, MA, USA), according to the manufacturer’s instructions. In brief, the extracted proteins were quantified using a Bio-Rad protein analysis kit (Bio-Rad Laboratories, Hercules, CA, USA). Equal amounts of each protein (40 μg) were separated by sodium-dodecyl sulfate-polyacrylamide gel electrophoresis and the transferred to polyvinylidene fluoride membranes (Millipore, Bedford, MA, USA). The membranes were blocked with 5% bovine serum albumin (Sigma-Aldrich Chemical Co.) for 1 h in a mixture of Tris-buffered saline and Tween 20 (TBST) and probed with primary antibodies overnight at 4 °C. The primary antibodies against Nrf2, Keap1, poly(ADP-ribose) polymerase (PARP), Bax, Bcl-2, caspase-3 and lamin B were purchased from Santa Cruz Biotechnology, Inc. (Dallas, TX, USA). The antibody for phosphor (p)-Nrf2 was obtained from Abcam, Inc. (Cambridge, UK). The primary antibodies against the histone variant H2A.X (ãH2A.X) and p-ãH2A.X were purchased from Cell signaling Technology (Danvers, MA, USA). Anti-HO-1 and anti-actin antibodies were obtained from Calbiochem-Novabiochem Co. (San Diego, CA, USA) and Bioworld Technology, Inc. (St Louis Park, MN, USA), respectively. The membranes were then incubated with the appropriate secondary antibodies (Santa Cruz Biotechnology, Inc.), conjugated with horseradish peroxidase (HRP) for 2 h at room temperature and rinsed three times with PBS. The protein bands were visualized by incubating the membranes in an enhanced chemiluminescence (ECL) reagent (Amersham Biosciences, Westborough, MA, USA) according to the manufacturer’s instructions. 

### 2.9. Determination of 8-hydroxy-2’-deoxyguanosine (8-OHdG)

The BIOXYTECH^®^ 8-OHdG-EIA™ kit (OXIS Health Products Inc., Portland, OR, USA) was used for the quantitative measurement of the oxidative DNA damage. Briefly, after the necessary experimental treatment, the cellular DNA was isolated using a DNA extraction kit (iNtRON Biotechnology Inc., Sungnam, Republic of Korea), following the manufacturer’s protocol, and quantified. The quantity of 8-OHdG, a deoxyriboside form of 8-oxoguanine, in the DNA, was determined by a calculation on a standard curve measured at 450 nm absorbance using a microplate reader, according to the manufacturer’s instructions.

### 2.10. Comet Assay for DNA Damage

The alkaline comet assay was performed to assess the oxidative DNA damage in individual cells, as previously described [25]. After the respective treatments, the cells were detached from the culture surface, mixed with 0.75% low-melting agarose (LMA) and dropped gently onto a microscope slide precoated with a layer of 0.75% normal-melting agarose. After the gel was formed, the cover slip was removed and the cell suspension was mixed with the LMA. The slides were then immersed in a lysis solution (2.5 M NaCl, 100 mM Na-ethylenediaminetetraacetic acid (EDTA), 10 mM Tris, 1% Triton X-100, and 10% DMSO, pH 10) for 1 h at 4 °C and electrophoresed in an alkaline electrophoresis solution (300 mM NaOH, 10 mM Na-EDTA, pH 10) for 20 min at 4 °C to allow DNA unwinding under alkaline/neutral conditions by incubating for 20 min. Thereafter, electrophoresis was carried out in the same buffer for 20 min at 4 °C to draw the negatively-charged DNA toward the anode. After electrophoresis, the slides were rinsed gently three times with a neutralization buffer (0.4 M Tris-HCl, pH 7.5) for 10 min at 25 °C. The slides were stained with ethidium bromide (EtBr, 40 μg/mL; Sigma-Aldrich Chemical Co.) and were observed under a fluorescence microscope, at an excitation wavelength of 485 nm and an emission wavelength of 530 nm. All of the above steps were conducted under yellow light to prevent additional DNA damage.

### 2.11. Measurement of MMP (Δψm)

To measure the loss of MMP, the cells were collected and incubated in media containing 10 μM of 5,5′6,6′-tetrachloro-1,1′,3,3′-tetraethyl-imidacarbocyanine iodide (JC-1; Sigma-Aldrich Chemical Co.), which is a mitochondria-specific fluorescent dye, for 20 min at room temperature in darkness. After washing twice with PBS to remove unbound dye, the green (JC-1 monomers) and red (JC-1 aggregates) fluorescence ratio that monitored the proportion of mitochondrial depolarization was immediately acquired on a flow cytometer by following the manufacturer’s protocol.

### 2.12. Detection of ATP Levels

The levels of intracellular ATP were determined using a firefly-luciferase-based ATP bioluminescence assay kit (Roche Applied Science, Indianapolis, IN, USA) according to the manufacturer’s instructions. Briefly, cells cultured under various conditions were lysed with the provided lysis buffer, and the collected supernatants were mixed with an equal amount of luciferase agent, which catalyzed the light production from the ATP and luciferin. The emitted light was immediately measured using a microplate luminometer, and the ATP level was calculated according to the ATP standard curve. The intracellular ATP levels were calculated as a percentage of the untreated control.

### 2.13. Colorimetric Assay of Caspase-3 Activity

Caspase-3 activities were assessed using a colorimetric assay kit (R&D Systems, Minneapolis, MN, USA). Briefly, collected cells were lysed after treatments, and equal amounts of proteins (150 μg) were incubated with the supplied reaction buffer, which contained the caspase-3 substrates dithiothreitol and tetrapeptides [Asp-Glu-Val-Asp (DEVD)-p-nitroaniline (pNA)], for 2 h at 37 °C in the dark. Changes in absorbance at 405 nm were determined using a microplate luminometer according to the manufacturer’s instructions. The results are presented as multiples of untreated control cell values.

### 2.14. Statistical Analysis

All experiments were performed at least three times. The data were analyzed using GraphPad Prism software (version 5.03; GraphPad Software, Inc., La Jolla, CA, USA), and expressed as the mean ± standard deviation (SD). Differences between groups were assessed using an analysis of variance followed by ANOVA-Tukey’s test, and *p* < 0.05 was considered to indicate a statistically significant difference. 

## 3. Results

### 3.1. Glutathione Inhibits H_2_O_2_-Induced Cytotoxicity in RAW 264.7 Cells

**Figure 1:**
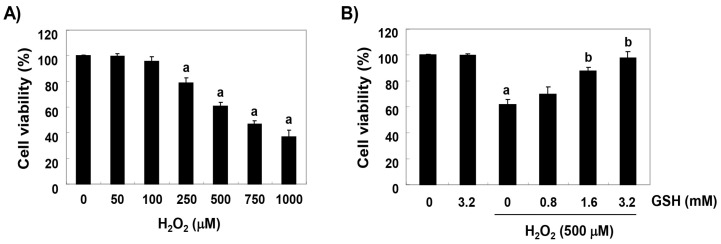
The protective effects of glutathione against the H_2_O_2_-induced cytotoxicity in RAW 264.7 cells. Cells were treated with (**A**) various concentrations of glutathione (GSH) for 24 h or (**B**) 500 μM H_2_O_2_ for 24 h following a 1 h glutathione pre-treatment with the indicated concentrations. After treatment, cell viability was examined by MTT assay. Data were expressed as the mean ± SD of three independent experiments (^a^ *p* < 0.05 compared with the control group; ^b^ *p* < 0.05 compared with the H_2_O_2_-treated group).

To establish the experimental conditions, RAW 264.7 cells were treated with various concentrations of H_2_O_2_ for 24 h, and the MTT assay was performed. As shown in Figure 1A, RAW 264.7 cells treated with concentrations of 200 μM or more showed a significant decrease in cell viability, but there was no significant change to the glutathione treatment up to 1 mg/mL compared with the control group (data not shown). Therefore, the H_2_O_2_ concentration for inducing oxidative stress was selected to be 500 μM, which showed a survival rate of about 60% compared with the untreated control cells. To evaluate the protective effect of glutathione on cytotoxicity induced by H_2_O_2_, RAW 264.7 cells were treated with 0.8 mM, 1.6 mM, and 3.2 mM glutathione for 1 h before a treatment with 500 μM H_2_O_2,_ after which the cells were cultured for 24 h. As shown in Figure 1B, the pretreatment with glutathione significantly restored the cell viability, as compared to H_2_O_2_ alone, in a concentration-dependent manner.

### 3.2. Glutathione Suppresses H_2_O_2_-Induced Apoptosis in RAW 264.7 Cells

**Figure 2:**
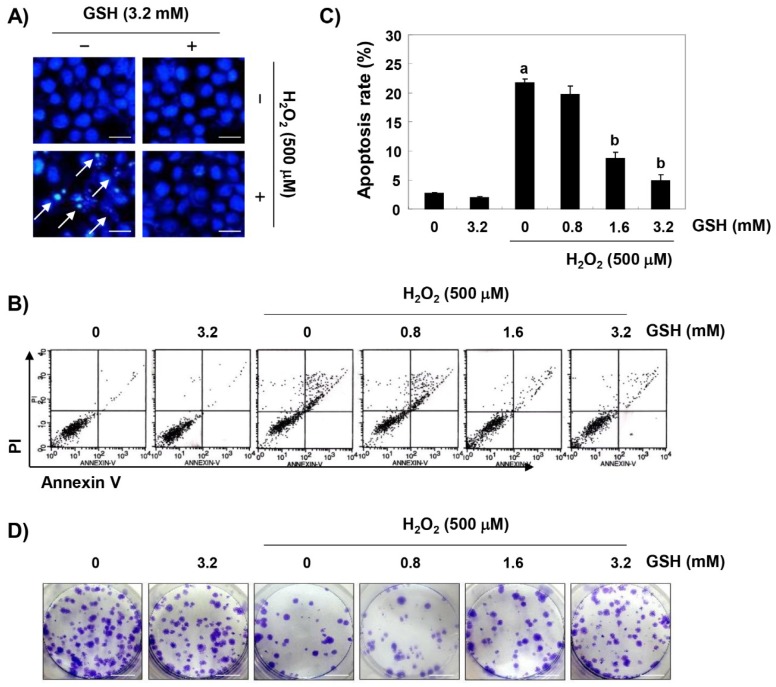
The inhibitory effects of glutathione against H_2_O_2_-induced apoptosis in RAW 264.7 cells. Cells were pretreated with the indicated concentrations of glutathione for 1 h and then stimulated with or without 500 μM H_2_O_2_ for 24 h. (**A**) The cells were fixed and stained with 4,6-diamidino-2-phenylindole (DAPI) solution, and the stained nuclei were pictured under a fluorescence microscope (original magnification, ×400). Representative photographs are shown. Scale bar, 50 µm. (**B**,**C**) The cells were stained with fluorescein isothiocyanate (FITC)-conjugated annexin V and propidium iodide (PI) for a flow cytometry analysis. (**B**) The results showed necrosis, defined as annexin V-negative and PI-positive cells (lower upper quadrant), early apoptosis, defined as annexin V-positive and PI-negative cells (lower right quadrant), and late apoptosis, defined as annexin V-positive and PI-positive (upper right quadrant) cells. (**C**) The percentages of apoptotic cells were determined by expressing the numbers of Annexin V-positive cells as percentages of all the present cells. The results are presented as the means ± SD of three independent experiments (^a^ *p* < 0.05 compared with the control group; ^b^ *p* < 0.05 compared with the H_2_O_2_-treated group). (**D**) After treatment, the cells were further cultured for two weeks to form colonies, then stained with a 0.1% purple-violet solution and imaged under inverted microscopy. Representative photographs are shown. Scale bar, 5 cm.

DAPI staining and flow cytometry analyses were performed to investigate whether the cytoprotective effect of glutathione against H_2_O_2_ on RAW 264.7 cells was related to the suppression of apoptosis. The fluorescent images in Figure 2A reveal that the control cells had intact nuclei, while the H_2_O_2_-treated cells showed significant chromatin condensation (see arrows). However, morphological changes were markedly attenuated in the cells pretreated with glutathione before the treatment with H_2_O_2_. The results of the annexin V/PI double staining also showed that the pretreatment with glutathione significantly decreased the frequency of apoptosis in H_2_O_2_-stimulated cells (Figure 2B). In addition, in cells treated with glutathione prior to H_2_O_2_ stimulation, the H_2_O_2_-induced colony formation was markedly reduced in a concentration-dependent manner (Figure 2C).

### 3.3. Glutathione Activates the Nrf2/HO-1 Signaling Pathway in H_2_O_2_-Treated RAW 264.7 Cells

**Figure 3:**
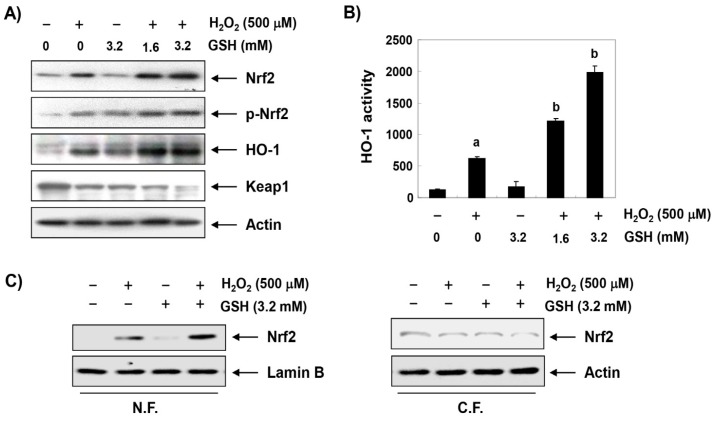
Effects of glutathione on the expression of Nrf2 and HO-1 in H_2_O_2_-treated RAW 264.7 cells. RAW 264.7 cells were pretreated with the indicated concentrations of glutathione for 1 h and then stimulated with or without 500 μM H_2_O_2_ for 1 h. (**A**) Western blot analyses were performed with the indicated antibodies. The proteins were visualized using an enhanced chemiluminescence (ECL) detection system. Actin was used as an internal control. (**B**) The nuclear and cytosolic proteins were prepared and followed by Western blotting using the indicated antibodies. Lamin B and actin were used as internal controls. N.F., nuclear fraction; C.F., cytosolic fraction. (**C**) The HO-1 activities of cells grown under the same conditions were determined based on the bilirubin formation. The data were shown as the mean ± SD obtained from three independent experiments (^a^ *p* < 0.05 compared with the control group; ^b^ *p* < 0.05 compared with the H_2_O_2_-treated group).

To investigate whether the anti-apoptotic activity of glutathione correlated with the activation of Nrf2/HO-1 signaling, the effect of glutathione on the expression of Nrf2 and its regulated gene HO-1 was determined. Our immunoblotting results showed that the expression of the HO-1 protein was not increased in the group treated with only glutathione at 1 mg/mL, but that it was slightly increased in the H_2_O_2_ only treatment group (Figure 3A). However, the expression of HO-1 in H_2_O_2_-treated cells was significantly increased by the treatment with glutathione compared to the cells treated with H_2_O_2_ alone (Figure 3A), and its enzymatic activity was also significantly increased (Figure 3B). In addition, the enhanced expression and activity of HO-1 by glutathione was associated with an increase in the total protein expression of Nrf2 and its phosphorylation at serine 40 (p-Nrf2), whereas the expression of Keap1, a negative regulator of Nrf2, was reduced in a glutathione treatment concentration-dependent manner (Figure 3A). Moreover, the increased Nrf2 expression in response to the H_2_O_2_ and/or glutathione treatment was translocated from the cytoplasm to the nucleus (Figure 3C).

### 3.4. The Inhibition of H_2_O_2_-Induced ROS Generation by Glutathione is Attenuated by ZnPP in RAW 264.7 Cells

**Figure 4:**
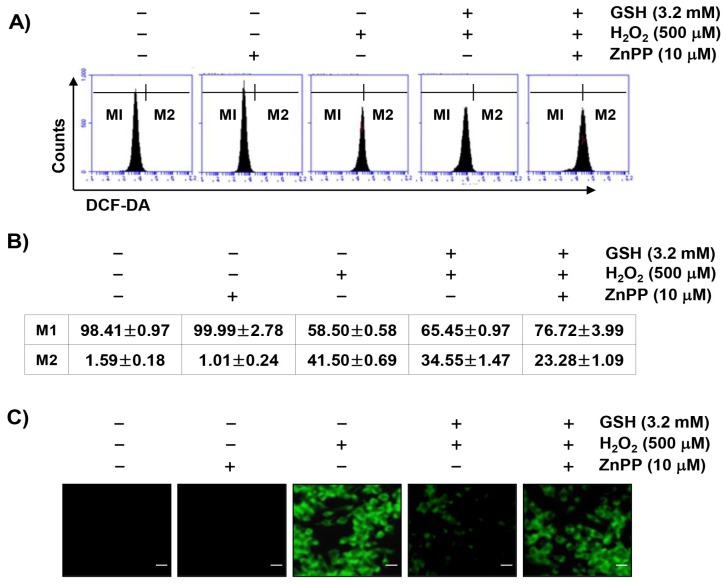
Inhibition of H_2_O_2_-induced ROS generation by glutathione in RAW 264.7 cells. Cells were pretreated with 3.2 mM glutathione or 10 μM ZnPP for 1 h and then treated with 500 μM H_2_O_2_ for 1 h. (**A**) After staining with 2′,7′-dichlorofluorescein diacetate (DCF-DA) fluorescent dye, DCF fluorescence was monitored by flow cytometer and fluorescent signals were displayed as histograms. (**B**) M1 is placed around the cells where no ROS is generated. M2 represents the percentage of cells that increase the reactive oxygen species (ROS) production. The data were shown as the mean ± SD obtained from three independent experiments. (**C**) Images were obtained by a fluorescence microscope (original magnification, ×200). Images are representative of at least three independent experiments. Scale bar, 20 µm.

We next investigated whether the protective effects of glutathione on the H_2_O_2_-induced cytotoxicity were due to a blockade of oxidative stress. As presented in Figure 4A,B, the production of ROS was markedly increased within 1 h in RAW 264.7 cells exposed to H_2_O_2_; however, the accumulation of ROS in the cells pretreated with glutathione was significantly reduced compared to the H_2_O_2_-only treatment. In the fluorescence microscope observations, we further confirmed that glutathione had a strong ROS scavenging effect (Figure 4C). Additionally, in order to determine whether glutathione-induced HO-1 activation was involved in the antioxidant activity of glutathione, RAW 264.7 cells were pretreated with ZnPP, an inhibitor of HO-1, and glutathione before being exposed to H_2_O_2_. As shown in Figure 4, ZnPP abrogated the protective effects of glutathione on the H_2_O_2_-induced ROS production, indicating that the cytoprotective effects of glutathione on oxidative stress may be mediated by a ROS generation blockade through HO-1 activation.

### 3.5. The Blockade of H_2_O_2_-Induced DNA Damage by Glutathione is Reduced by ZnPP in RAW 264.7 Cells

**Figure 5:**
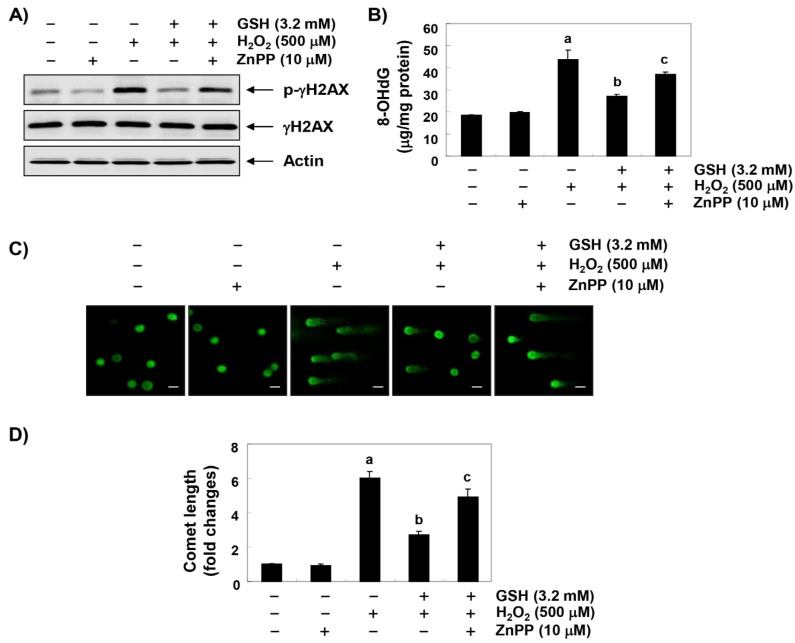
Protection against H_2_O_2_-induced DNA damage by glutathione in RAW 264.7 cells. Cells were pretreated with 3.2 mM glutathione or 10 μM ZnPP for 1 h and then stimulated with or without 500 mM H_2_O_2_ for 24 h. (**A**) The cellular proteins were prepared, and p-γH2AX and γH2AX protein levels were assayed by Western blot analysis using an ECL detection system. Actin was used as an internal control. (**B**) The amount of 8-OHdG in the DNA was determined using an 8-OHdG-EIA kit. The measurements were made in triplicate, and values are expressed as the mean ± SD (^a^ *p* < 0.05 compared with the control group; ^b^ *p* < 0.05 compared with the H_2_O_2_-treated group; and ^c^ *p* < 0.05 compared with the glutathione- and H_2_O_2_-treated group). (**C**,**D**) The comet assay was performed, representative images of the comet assay were taken by a fluorescence microscope (original magnification, ×200, Scale bar, 40 µm) and the comet length (fold of control) was quantified (^a^ *p* < 0.05 compared with the control group; ^b^ *p* < 0.05 compared with the H_2_O_2_-treated group; and ^c^ *p* < 0.05 compared with the glutathione- and H_2_O_2_-treated group).

We subsequently performed three assays to determine whether glutathione prevents DNA damage. Immunoblotting results showed a marked increase in γH2AX phosphorylation (p-γH2AX, at serine 139), one of the DNA strand break markers, in H_2_O_2_-stimulated cells compared to untreated control cells; however, the increased levels of p-γH2AX caused by H_2_O_2_ were suppressed almost to control levels in the presence of glutathione (Figure 5A). In addition, the H_2_O_2_ treatment significantly increased the production of the 8-OHdG adduct, an oxidative stress-induced DNA damage marker, compared to the control group, but the pretreatment with glutathione significantly reduced the production of 8-OHdG by H_2_O_2_ (Figure 5B). Furthermore, in the comet assay, another method for detecting DNA strand breaks, there was no smeared pattern of nuclear DNA in the untreated controls and the cells treated only with glutathione. However, in the H_2_O_2_-treated cells, the length of the comet tail clearly increased, which means that DNA damage occurred; and in glutathione pretreated cells, the tail length was obviously shorter than in the H_2_O_2_-treated cells (Figure 5C,D). On the other hand, ZnPP excluded some, but not all, of the protective effects of glutathione against H_2_O_2_-induced DNA damage, suggesting that the cytoprotective effect of glutathione on oxidative stress in RAW 264.7 cells is partially mediated through HO-1 induction.

### 3.6. The Reduction of H_2_O_2_-Induced Mitochondrial Dysfunction by Glutathione is Diminished by ZnPP in RAW 264.7 Cells

**Figure 6:**
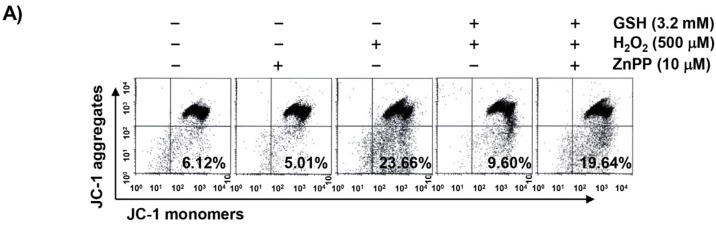

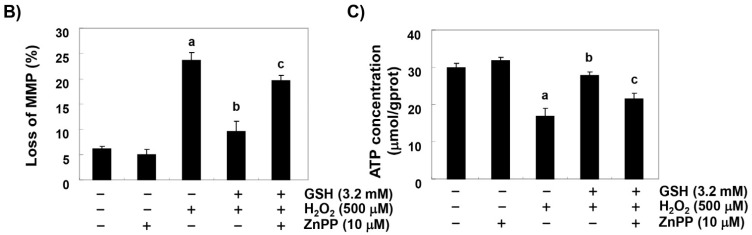
Attenuation of H_2_O_2_-induced mitochondrial dysfunction by glutathione in RAW 264.7 cells. Cells were pretreated with 3.2 mM glutathione or 10 μM ZnPP for 1 h and then stimulated with or without 500 μM H_2_O_2_ for 24 h. (**A**) The cells were collected and incubated with 10 µM JC-1, and the MMP values were obtained with a flow cytometer. The top right quadrant indicates normal mitochondria, whereas the bottom right quadrant indicates depolarized mitochondria. (**B**) The results are the mean ± SD obtained from three independent experiments. (**C**) To monitor the ATP production using a luminometer, a commercially available kit was used. Each point represents the mean ± SD of three independent experiments (^a^ *p* < 0.05 compared with the control group; ^b^ *p* < 0.05 compared with the H_2_O_2_-treated group; and ^c^ *p* < 0.05 compared with the glutathione- and H_2_O_2_-treated group).

In order to examine the protective effect of glutathione on mitochondrial dysfunction caused by H_2_O_2_, MMP and intracellular ATP levels were evaluated. According to the results of JC-1 staining shown in Figure 6A,B, changes in the ratio of polarized and depolarized cell populations were observed in RAW 264.7 cells treated with H_2_O_2_, and the increase in the depolarized mitochondrial membrane was about 4 times higher than in the control group. Along with these results, the concentration of ATP in cells exposed to H_2_O_2_ was significantly decreased compared with cells cultured in the normal medium (Figure 6C). Glutathione was able to prevent these changes, but the protective effects of glutathione were significantly abrogated in the presence of ZnPP, demonstrating that the activation of HO-1 was involved in this protective activity.

### 3.7. The Inhibitory Effect of Glutathione on the Change of Apoptosis Markers Genes by H_2_O_2_ is Reversed by ZnPP in RAW 264.7 Cells

**Figure 7:**
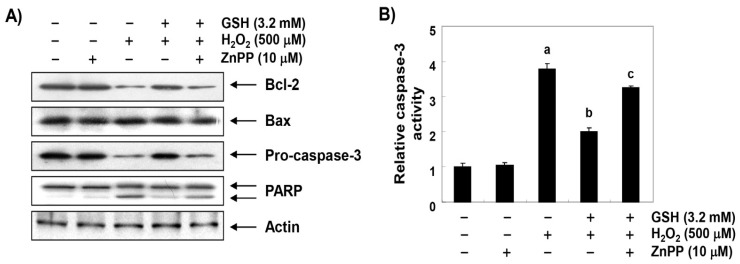

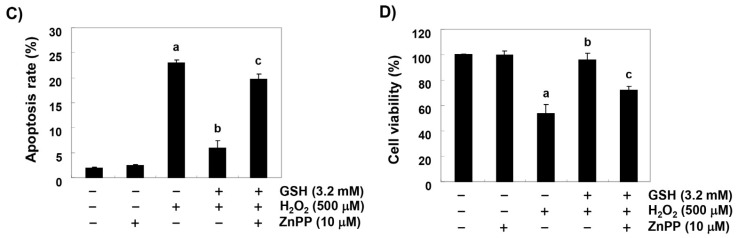
Effects of glutathione on the expression of apoptosis regulatory genes in H_2_O_2_-treated RAW 264.7 cells. (**A**) After a treatment with 3.2 mM glutathione or 10 μM ZnPP in the presence or absence of H_2_O_2_ for 24 h, the cellular proteins were prepared, and the protein levels were assayed by Western blot analysis using an ECL detection system. Actin was used as an internal control. (**B**) The cells were lysed, and aliquots were assayed for in vitro caspase-3 activity using DEVD-pNA as substrates. The amount of pNA released was measured at 405 nm using an ELISA microplate reader. (**C**) To quantify the degree of apoptosis, the cells were stained with FITC-conjugated annexin V and PI for a flow cytometric analysis. (**D**) Cell viability was examined by MTT assay. The results are the mean ± SD obtained from three independent experiments (^a^ *p* < 0.05 compared with the control group; ^b^ *p* < 0.05 compared with the H_2_O_2_-treated group; and ^c^ *p* < 0.05 compared with the glutathione- and H_2_O_2_-treated group).

To further investigate the molecular mechanisms of the apoptosis-protective effect of glutathione and the role of HO-1, we examined the effects of glutathione and ZnPP on H_2_O_2_-induced changes of the apoptosis-regulated gene expression. The immunoblotting results shown in Figure 7A demonstrate that the anti-apoptotic Bcl-2 protein was significantly down-regulated in H_2_O_2_-treated RAW 264.7 cells, while the pro-apoptotic Bax protein remained unchanged. Additionally, the expression of pro-caspase-3 was markedly reduced, and its activity was increased about 4 times in the H_2_O_2_-treated cells as compared with the control. In addition, the expression of cleaved PARP, a representative substrate protein degraded by activated caspase-3, was also increased (Figure 7A,B). These changes resulting from the H_2_O_2_ treatment were relatively conservative in the cells that were pretreated with glutathione, but the protective potentials of glutathione disappeared under the condition in which the activation of HO-1 was suppressed. It is noteworthy that ZnPP also excluded the beneficial effects of glutathione on H_2_O_2_-induced apoptosis and survival reduction (Figure 7C,D), indicating that the protective role of glutathione was at least somewhat dependent on HO-1.

## 4. Discussion

Glutathione is an endogenously produced non-enzymatic antioxidant that plays a critical role in regulating intracellular redox-sensitive signal transduction [10,11,12,13,14,17]. In particular, the depletion of glutathione has been proposed as an early event prior to the initiation of apoptosis by oxidants inducing the ROS production, while the supplementation of glutathione has the effect of preventing such apoptosis [15,16,18]. However, the role of Nrf2/HO-1 signaling in relation to the cytoprotective effect of glutathione against oxidative stress has not been well studied. Therefore, in the present study, we investigated whether glutathione is effective in preventing oxidative stress-induced cytotoxicity through Nrf2-mediated HO-1 activation. The results of the present study demonstrated that the supplementation of exogenous glutathione prevented H_2_O_2_-induced apoptosis through the rescue of the mitochondrial function by blocking the ROS accumulation. It was also found that glutathione promoted the activation of the Nrf2/HO-1 signaling pathway, and that the inhibition of the HO-1 activity eliminated the protective effect of glutathione, suggesting that the protective effects of glutathione in RAW 264.7 cells were, at least, HO-1 dependent.

As is well known, the mechanism of Nrf2 induction is dependent on the inducers and cell types, but Nrf2 plays a central role in protecting cells from oxidative damage by regulating the transcriptional activity of antioxidant genes, including HO-1 [19,20]. Similar to the results of this study, previous studies have shown that H_2_O_2_ can enhance the expression of HO-1 [26,27], and the up-regulation of HO-1 has been identified as a defense mechanism against H_2_O_2_-induced apoptosis in a variety of cell types [22,26]. The overexpression of HO-1 has also exhibited resistance to DNA damage and apoptosis induced by oxidative stress [20,23,28]; however, the inhibition of HO-1 activity improved cytotoxicity against oxidative stress and reduced the efficacy of antioxidants [29,30]. Our results show that H_2_O_2_ alone partially increases the expression of HO-1 as well as the expression of Nrf2, but the expression of both proteins and the activity of HO-1 were further markedly increased by the co-treatment with glutathione when compared to cells treated with H_2_O_2_ alone. In addition, the expression of p-Nrf2, an active form of Nrf2, was also significantly increased under the same conditions, which was related to the nuclear translocation of Nrf2. Therefore, we hypothesized that the induction of HO-1 by glutathione could block DNA damage and the apoptosis of RAW 264.7 cells caused by oxidative stress by blocking ROS generation. In this study, we also investigated the effects of ZnPP, an HO-1 specific inhibitor, on the inhibitory effect of glutathione on the ROS production in H_2_O_2_-treated cells and observed that the inhibition of the ROS production by glutathione was abolished by the ZnPP treatment. These results suggest that the protective effect of glutathione on H_2_O_2_-induced oxidative stress is mediated through the activation of Nrf2/HO-1 signaling. Furthermore, the beneficial effect of glutathione on H_2_O_2_-induced DNA damage was significantly reduced by ZnPP, which also implies that the protective role of glutathione on H_2_O_2_-induced cytotoxicity was dependent on HO-1.

Most experimental evidence supports the role of mitochondria as the main target intracellular organelle for H_2_O_2_ toxicity, and excessive ROS accumulation due to oxidative stress is one of the mechanisms leading to apoptosis associated with mitochondrial injury [1,2]. In addition, the activation of HO-1 may also lead to the inactivation of the mitochondria-mediated intrinsic apoptosis pathway. It has been recognized as a cell protection mechanism for oxidative stress-mediated mitochondrial dysfunction [20,23]. In the induction of ROS-mediated apoptosis, ROS overload causes the free radical attack of the membrane phospholipid, which in turn leads to a mitochondrial membrane depolarization resulting in the loss of MMP. This is considered to be the onset of the intrinsic apoptosis pathway [4,31]. At the same time, mitochondrial dysfunction promotes abnormalities in the mitochondrial respiratory chain’s electron transport pathways, ultimately interfering with the intracellular ATP production [5,32]. Consistent with previous studies [33,34,35], current studies have shown that when cells are exposed to H_2_O_2_, the MMP levels and ATP content are significantly reduced compared to controls, whereas the co-treatment with glutathione significantly reverses the H_2_O_2_-induced loss of MMP and APT. However, in the presence of ZnPP, the glutathione-mediated repair of the mitochondrial dysfunction and decreased production of ATP were significantly diminished. In view of maintaining energy homeostasis, these results are in good agreement with previous studies showing that the protective effects of apoptosis against oxidative stress are related to the maintenance of the ATP production through the preservation of the mitochondrial function [36,37,38]. We therefore consider that the conservation of the ATP production due to the retention of the mitochondrial function is one possible mechanism by which glutathione can preserve the cell survival pathway from oxidative stress through HO-1 activation.

The activation of caspase-9 by the release of apoptotic factors, including cytochrome *c*, from the mitochondria to the cytoplasm due to the loss of MMP is a major initial step in the initiation of a caspase-dependent intrinsic apoptosis pathway [39,40]. Also, the activation of caspase-9 ultimately activates downstream effector caspases, including caspase-3 and caspase-7, eventually leading to cell death. This process is accompanied by the degradation of the substrate proteins of effector caspases such as PARP, and the fragmentation of these proteins is used as evidence that caspase-dependent apoptosis is induced [39,41]. The activation of caspase is regulated by various proteins, including Bcl-2 family members consisting of anti-apoptotic and pro-apoptotic proteins. Among the Bcl-2 family members, anti-apoptotic proteins such as Bcl-2 are located on the outer mitochondrial membrane to prevent the release of apoptogenic factors and provide protection by inhibiting the consumption of ATP [41,42]. On the other hand, pro-apoptotic proteins, including Bax, antagonize anti-apoptotic proteins or translocate to mitochondrial membranes to form membrane-integrated homo-oligomers that induce mitochondrial pore formation, leading to the loss of MMP and resulting in the cytosolic release of apoptotic factors [43,44]. Therefore, the balance of apoptotic Bax family proteins to the anti-apoptotic Bcl-2 family proteins serves as a determinant of the induction or inhibition of the activation of the caspase cascade for the initiation of the intrinsic apoptosis pathway. Many previous studies have shown that the induction of apoptosis by H_2_O_2_ in RAW 264.7 cells was associated with a decrease in the Bcl-2/Bax ratio and/or activation of caspases [45,46]. However, several natural antioxidant products that can protect against H_2_O_2_-mediated cytotoxicity have altered this tendency [47,48]. Consistent with previous findings, our results showed that the decreased expression of Bcl-2 in H_2_O_2_-treated RAW 264.7 cells was reverted in the presence of glutathione. In addition, the H_2_O_2_-induced activation of caspase-3 and the degradation of PARP were also blocked by glutathione administration concomitant with increased cell viability and reduced apoptosis. In this respect, it is suggested that glutathione can rescue H_2_O_2_-induced cytotoxic injury in RAW 264.7 cells by blocking mitochondria-mediated oxidative stress and apoptosis. However, these protective effects of glutathione against H_2_O_2_ were markedly hindered by the inhibition of the HO-1 function by an HO-1 inhibitor. These results are in good agreement with other recent studies showing that HO-1 restored cell survival through the prevention of oxidative damage-mediated apoptosis [34,49,50]. Therefore, although further studies on the relevance of multiple GSH-dependent enzymes should be undertaken, the current results indicate that the cellular protective potential of glutathione against oxidative stress in RAW 264.7 cells is at least dependent on the activation of Nrf2/HO-1 signaling.

## 5. Conclusions

In conclusion, the present study confirms that exogenous glutathione can effectively prevent RAW 264.7 cells from H_2_O_2_-induced cytotoxicity by blocking oxidative stress-mediated DNA damage and the mitochondria-dependent apoptotic pathway, which was associated with the activation of Nrf2/HO-1 signaling. Although studies on mitochondrial damage-associated energy metabolism and glutathione downstream signal molecules are needed, these findings may be presented as additional evidence that the intracellular uptake of glutathione can alter the redox state of cells and thereby regulate cellular antioxidant signaling pathways.
